# Impact of Simulated Microgravity on Cytoskeleton and Viscoelastic Properties of Endothelial Cell

**DOI:** 10.1038/srep32418

**Published:** 2016-09-01

**Authors:** M. Janmaleki, M. Pachenari, S. M. Seyedpour, R. Shahghadami, A. Sanati-Nezhad

**Affiliations:** 1 BioMEMS and Bioinspired Microfluidic Laboratory, Center for BioEngineering Research and Education, Department of Mechanical and Manufacturing Engineering, University of Calgary, Canada; 2 Medical Nanotechnology and Tissue Engineering Research Center, Shahid Beheshti University of Medical Sciences, Tehran, Iran; 3 Chair of Mechanics - Structural Analysis - Dynamics, Faculty of Architecture and Civil Engineering, TU Dortmund, Germany; 4 Department of Medical Physics and Biomedical Engineering, Shahid Beheshti University of Medical Sciences, Tehran, Iran

## Abstract

This study focused on the effects of simulated microgravity (s-μg) on mechanical properties, major cytoskeleton biopolymers, and morphology of endothelial cells (ECs). The structural and functional integrity of ECs are vital to regulate vascular homeostasis and prevent atherosclerosis. Furthermore, these highly gravity sensitive cells play a key role in pathogenesis of many diseases. In this research, impacts of s-μg on mechanical behavior of human umbilical vein endothelial cells were investigated by utilizing a three-dimensional random positioning machine (3D-RPM). Results revealed a considerable drop in cell stiffness and viscosity after 24 hrs of being subjected to weightlessness. Cortical rigidity experienced relatively immediate and significant decline comparing to the stiffness of whole cell body. The cells became rounded in morphology while western blot analysis showed reduction of the main cytoskeletal components. Moreover, fluorescence staining confirmed disorganization of both actin filaments and microtubules (MTs). The results were compared statistically among test and control groups and it was concluded that s-μg led to a significant alteration in mechanical behavior of ECs due to remodeling of cell cytoskeleton.

Microgravity condition leads to endothelium dysfunction which in turn causes the individuals to experience cardiovascular deconditioning as well as physiologic changes^1^. Gravitational alterations influence endothelial cell (EC) proliferation, differentiation, signaling, gene expression, surface adhesion molecules, extracellular matrix proteins expression, and cause significant changes in cytoskeletal polymers^2^,^3^,^4^,^5^,^6^,^7^,^8^,^9^,^10^.

Endothelium plays a crucial role in local blood flow, regulation of coagulation, permeability, leukocyte adhesion, and vascular smooth muscle cell growth. The structural and functional integrity of the ECs are vital to regulate vascular homeostasis and prevent atherosclerosis^11^,^12^. Chemical regulators, extracellular matrix, and environmental conditions adjust the cells’ functions. Mechanical loadings such as shear stresses resulted from blood flow, hydrostatic pressure, and cyclic stretch are some examples of environmental stimuli able to alter the functionality and mechanical properties of ECs^13^,^14^. In addition to regulatory effects of such stresses, in critical conditions, the extreme values may initiate pathologic problems such as atherosclerosis and intima hyperplasia^15^. DNA microarray analysis revealed that about 600 genes (3% of all genes) of ECs responded to shear stress^16^. On the other hand, altered mechanical properties of cells may contribute to cell remodeling and regulation of stresses within the cell body^17^. Actin filaments align to the direction of shear stress, which consequently lead the cells spindle-shaped. Cells experiencing turbulent flow found a rounder shape with non-uniform orientations^18^,^19^.

It has been demonstrated that mechanical unloading (MU) remodeled actin cytoskeleton polymer, reduced the total amount of this biopolymer through transcriptional mechanism^4^,^20^, and had a significant effect on the arrangement and dynamics of microtubules^21^. The detailed mechanisms involved in conversion of MU to intracellular biochemical reactions are still elusive. Therefore, *in vitro* studying of the endothelial behavior in response to microgravity conditions in term of mechanical properties assists in understanding of endothelium alterations, mechanisms involved in vascular problems, and pathogenesis of related diseases in space mission. Several studies have been done to evaluate the mechanical properties of cells exposed to a variety of mechanical stresses by using either quantitative and qualitative techniques^22^. Micropipette aspiration (MA) is the most feasible method, which enables measurement of the whole cell mechanical properties. It eliminates disadvantageous effects of cell-matrix interaction and more importantly the stiffness of substrate^23^,^24^.

The goal of this study was to investigate the impact of s-μg on viscoelastic parameters of ECs and the content of main cytoskeleton polymers. A developed RPM was utilized to simulate weightless conditions by means of continuous random change of orientation, relative to the gravity vector (Fig. 1)^25^. Concerning difficulties with space experiments, RPM is an appropriate candidate for ground-based evaluations with a reliable performance comparing to real microgravity^26^,^27^,^28^. In this report, primary ECs were placed under s-μg conditions by which viscoelastic properties, proliferation, morphology, and cytoskeleton content were investigated. To the best of our knowledge, this work is the very first study on the effects of microgravity on the cell mechanical properties and the linkage with the main cytoskeletal polymers contents.

## Results

### Mechanical properties of ECs

All ECs had typical viscoelastic solid creep behavior responding to a 600 Pa step pull-in pressure. Deformation (*L*) and time (*t*) had a nonlinear regression with a mean of R^2^ = 0.9781 ± 0.017 for all cells. The average diameters of EC cells were measured in the range of 12–16.8 μm. In Fig. 2 a real image of MA experiment for an endothelial cell after 72 hrs of being subjected to unloading is illustrated. Estimated viscoelastic parameters are shown in Fig. 3. There was a considerable reduction in the measured elasticity and the viscosity for cells under microgravity. The results indicated statistically significant differences in *K*_*1*_ within 24 hrs and later (*p* < *0.01*). The mean Young’s modulus of elasticity (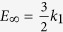) for normal ECs and the cells under microgravity at 12, 24, 48, and 72 hrs were obtained to be 129.62 ± 19, 122.88 ± 12, 101.46 ± 13, 62.15 ± 9, and 32.21 ± 6 Pa, respectively (Fig. 4). A similar declined trend was observed for the viscosity (*μ*) with no statistically significant drop at 12 hrs (*p* > *0.22*). The time constant (*τ*) reduced at the selected time points; however, the reduction was statistically meaningful at 48 hrs and 72 hrs (*p* < *0.04*). The instantaneous Young’s modulus (
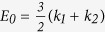
) decreased significantly (*p* < *0.001*), where the values at 24 hrs and 72 hrs were approximately 30% and 60% of the control cells, respectively. No significant correlations were observed between the diameters and the viscoelastic parameters of cells (*p* > *0.08*).

### Circularity

Circularity was defined as the ratio of width to length of each cell. Endothelial morphology changed from a normal spindle shape (Fig. 5a) to a cobblestone phenotype (Fig. 5b). It is obvious that the value increased under s-μg. After 24 hrs of being unloaded, circularity increased about 3.3 times in comparison with control cells (Fig. 5c). The average surface area of ECs was also measured using ImageJ which showed an increase of 43% under s-μg. 4 different petri dishes with minimum cell number of 200 cells were evaluated for circularity measurement (*p* < *0.05*).

### Cell proliferation assay

The MTT (3-(4, 5-dimethylthiazol-2-yl) 2, 5-diphenyl tetrazolium bromide) assay was used to evaluate the effect of microgravity on ECs viability at 24 hrs. Results confirmed slight changes in cell populations (Fig. 5d). Being exposed under s-μg caused an average drop of 7% in cells’ viability. The reduction for cells under s-μg is not statistically insignificant (n = 6, p > 0.05) comparing to control cells.

### Cytoskeleton Content and staining

Western Blot (WB) analysis was performed to estimate the content of F-actins and MTs in ECs. The results helped to quantify the alterations of two main cytoskeletal biopolymers. Figure 6 shows the densitometric quantization of the relative band intensities for cells under 24 hrs of being subjected to microgravity and control cells. It was clear that the expression of both cytoskeleton filaments is reduced. The content of F-actins protein reduced around 65% and the reduction for β-tubulin expression was 26% under the microgravity simulation. Similarly, fluorescent staining revealed a considerable disruption in structural filamentous polymers. Figure 7 shows the MTs and the actin filaments of the cells for control and s-μg condition. The images show that the more cell being under simulated microgravity, the more the cytoskeleton disrupts. The actin rim underneath the plasma membrane was no more continuous. Microtubules network obviously disorganized and relatively concentrated around the nucleus.

## Discussion

Mechanical properties of EC provide the cell ability to resist widespread hemodynamic forces and at the same time, to respond differentially to variations in different surrounding stresses^29^. ECs constantly experience shear stress, cyclic stretch, and hydrostatic pressure. The developed mechanical stresses within the cell body have shown an evident role in the endothelium function. Furthermore, altered mechanical properties of cells by microenvironmental loadings have influenced cell remodeling and regulation of the stresses^17^. Vascular endothelium dysfunction undoubtedly influences vascular smooth muscle proliferation, angiogenesis, and wound healing. It is generally believed that the endothelial mechanical properties, gravisensation, and gravitropism are mostly defined by the cytoskeleton. The adaptation of cell mechanical tension to any changes in external mechanical stresses depends on the cell mechanical characteristics and the sensitivity of mechanosensors^30^. Similar to other eukaryotic cells, the endothelial cytoskeleton strongly depend on three major entangled filamentous biopolymers: actin filaments, microtubules, and to a less extent intermediate filaments. This highly dynamic network defines cell shape, withstand external forces, and effectively respond to mechanical stimuli^29^. Actin filaments, besides structural responsibility, play key functions in cell morphology, migration, phagocytosis, vesicular movement, cytokinesis, and molecular transport between the plasma membrane and the nucleus^31^,^32^. Cell locomotion is mediated by actin networks in cell cortex. While F-actins resist against deformations, they fluidize under high shear stresses. They respond to external forces and they have substantial effect in formation of leading-edge protrusions during cell motility^33^. Therefore, any disorganization of actin filaments may interfere with the transmission of forces across the cell membrane^34^. Microtubules are essential skeletal elements in cell division, polarization, and migration. Microtubules as the second major constituent of the cytoskeleton act in concert with the other filament biopolymers to stabilize the cell structure under compression loadings. According to the tensegrity theory, MTs as load-bearing compression elements and actin filaments as tension elements support integrity of cells^35^. ECs showed sensitivity to short term microgravity (22 s) in rearrangement of their β-tubulin^5^. Moreover, being subjected to s-μg more than 96 hrs caused a decrease in the total amount of actin as well as disorganization of actin cytoskeleton which could lead to apoptosis. In one word, the responses of cells to microgravity are cell-type and exposure-time dependent^4^.

In this study, we demonstrated that microgravity could change mechanical properties, content of the main cytoskeleton polymers, and morphology of ECs. Previous studies confirmed viscoelastic behavior of ECs^14^,^36^. While deformation of cell body is mostly determined by elasticity, viscoelasticity characterizes time dependent alteration in deformation. Viscoelastic parameters of ECs were evaluated quantitatively by MA technique over 72 hrs. Young’s modulus and viscosity of the ECs decreased during microgravity experiments. As described by results of the current study, microgravity caused a significant increase in deformability of the cultured ECs. This effect was intensified by s-μg exposure time. Although mechanical parameters of ECs did not experience statistically meaningful reduction at 12 hrs, *K*_*1*_ and *μ* decreased substantially about 20% (p < 0.01) and 40% (p < 0.001) at 24 hrs and it continued reduction until 72 hrs. As the viscoelastic properties of the cells showed significant alterations at 24 hrs, this time point was selected to compare the cytoskeleton reorganization with control cells. Accordingly, MTT assay, western blotting were performed at that time point. There are a few studies that addressed a short period viability for ECs under microgravity and any possible consequences on the cell structure^4^,^11^. MTT assay was performed to make sure the least side effect of s-μg on cell survival at the time of cytoskeleton evaluation. Viability assay depicted a trivial effect of microgravity on ECs proliferation at 24 hrs. Subsequently, WB analysis and fluorescence staining were done at this time point. Although trypsin used for cell detachment may affect the cytoskeleton, the observed changes in mechanical properties are mainly generated by s-μg. All mechanically investigated cells were treated in similar conditions with similar detaching approach and MA. Trypsin’s concentration, volume, exposing time were maintained similar in all experiments, thereby the differences in mechanical properties were mostly associated with the microgravity exposure time. Disruption of stress and tensile fibers and orientation of filamentous bundles can be influential in both elastic and viscous parameters of cellular mechanical model. Although polymerization and depolymerization of the main cytoskeletal fibers are much faster comparing to continuous random change of orientation by RPM, it is shown in line with other reports that the total amount of actin filaments and microtubules decreased^8^,^19^,^37^,^38^,^39^,^40^. Western blot analysis, which was performed very immediately after simulated microgravity, confirmed a considerable reduction in content of both actin filaments and microtubules. Obviously, actin filaments were more susceptible to unloading by experiencing a dramatic drop of 65% comparing to 26% reduction in microtubules. It is believed that a drop in actin filaments is an adaptive mechanism to avoid the accumulation of redundant actin fibers^5^. This phenomena is likely due to transcriptional regulation of the cytoskeletal polymers^4^,^38^,^41^, where the down-regulation can lead to a lasting reduction in the synthesis of the cytoskeletal polymers. Cytoskeleton staining revealed disorganization of both actin filaments and microtubules. The more cells being under microgravity the more the cytoskeleton disrupts. Cell staining requires fixation using materials such as paraformaldehyde which helps to preserve cellular architecture and composition of cells. As the staining procedure started vey immediately after each s-μg experiment, the visualized cytoskeleton of the cells obviously proved the disorganization of actin filaments and microtubules caused by simulated microgravity. The amount of actin filaments particularly around the membrane was reduced but the reduction for microtubules was less considerable. In fact, the altered mechanical characteristics of ECs can be mostly attributed to the rearrangement and reduction in the content of cytoskeletal actin filaments. There was some time interval between sample dismounting and cells’ preparation for MA which might affect cytoskeleton structure however, it could not have a substantial contribution in alteration of mechanical properties among samples. The evaluated ECs were treated carefully in a same manner therefore, the differences between viscoelastic properties of the cells are stemmed from the effects of s-μg. More importantly, the values emphasized in this research are the differences in mechanical properties rather than absolute values. This means that even if there are some effects of delays or trypsin on cytoskeletal properties, we are still able to observe the effect of simulated microgravity on mechanical properties and cytoskeleton of cells, although this effect may not be linearly associated.

Shear stresses are transmitted to the cortical actin rim immediately underneath the plasma membrane of EC. Actin polymers form a cortical rim in quiescent ECs which provides a link between extracellular events and intercellular organelles^42^. Densely polymerized array of actin filaments underneath of membrane has the major role to reinforce membrane against intense cortical deformation^43^. The rigidity of the cortex as a fundamental property is vital in regulation of cell shape, cytokinesis, proliferation, differentiation, and the response to a change in external environment. In addition, formation of protrusive organelles such as filopodia, lamellipodia, and microvilli is regulated by very local changes in membrane rigidity^44^. Disruption of actin bundles suggested evaluating mechanical behaviors of EC cortex. MA experiment begins with the investigation of cell cortex and its mechanical properties can be estimated by the instantaneous Young modulus (*E*_*0*_). This parameter decreased approximately 20% at 12 hrs and reached 67% of its initial value at 24 hrs. On the other hand, E lessened insignificantly 5% at 12 hrs and the reduction was approximately 20% at 24 hrs of being in s-μg condition. At 48 hrs, the reduction of both E_0_ and E were similar and around 50% of their initial values. Finally at 72 hrs, reduction of Young’s modulus exceed instantaneous Young’s modulus. Indeed, E and E_0_ decreased approximately 75% and 60%, respectively, at 72 hrs. In other words, cell membrane stiffness was more susceptible to disruption than whole cell body stiffness at early hours of experiencing microgravity condition, whereas long term s-μg had more influence on whole cell rigidity than cortical rigidity. It is evident that disruption of cortical actin boosts endothelial permeability and stops barrier function of the cells^12^,^45^. Fluorescence staining suggested that the mechanical alterations of the cortical actin rim occurred because of extensive disruption of actin filaments. This has a significant impact on endothelial dysfunction and vascular damage.

Microgravity had effects on both cell shape and cytoskeleton. Image processing algorithms showed that ECs roundness increased under s-μg significantly. The length and width of the cells decreased in a manner that the circularity of the cells raised. This morphological alteration was a consequence of cytoskeletal reorganization at the subcellular level. The circularity parameter increased about 3 times after 24 hrs of s-μg, which is consistent with previous reports^8^,^46^. The imbalance of forces on a cell seemingly caused an extracellular matrix remodeling and consequently changes in morphology^46^,^47^. It is believed that cell rounding and, consequently, barrier integrity is mainly determined by the actin filaments organization. It was suggested that a reduction in cell-matrix attachment comparing to cytoskeletal arrangement and susceptibility of PKC-mediated signal transduction to microgravity caused the cells to be rounded in shape and not in spread^3^,^46^,^48^.

In conclusions, it is clearly demonstrated that there is a close relationship between mechanical properties, the main cytoskeletal polymer contents, and endothelial cell shape. The findings of this paper indicate an adaptive response of ECs to microgravity conditions through an alteration in cytoskeleton structure. Microgravity disrupts the polymerization and depolymerization processes of the main cytoskeleton polymers and consequently causes improper function by reducing cytoskeleton-generated tensions. Although s-μg and its effect on mechanical properties of ECs have been studied, the related mechanotransduction should be fully understood by further studies. These changes certainly have a direct influence on signal transduction, synthesis and secretion of cytokines, and gene expression, which can ultimately lead to apoptosis. Hence, an exact evaluation of the proteome and utilizing the gene array techniques may expand knowledge about the involved signaling pathways in endothelial dysfunction and mechanism involved in vascular disorders in microgravity conditions. This may eventually help to find novel approaches to prevent vascular disorders. Possibility of creating 3D differentiated tissue assemblies has attracted several researchers to culture cells in microgravity conditions^49^,^50^,^51^. Accordingly, alterations of the mechanical features under s-μg need to be known. In addition, the results from s-μg conditions on mechanical behaviors of ECs should be compared with real microgravity experience in space.

## Methods

### Cell culture

Human umbilical vein endothelial cells (HUVECs) were obtained from a commercial source (ATCC CRL-1730). Cells were maintained at 37 °C (5% CO_2_, 95% air) in Ham’s F12 + DMEM (1/1V), supplemented with 10% fetal bovine serum (Gibco), and 1% penicillin/streptomycin (Gibco). ECs were cultured on standard 35-mm diameter petri dishes at a density of 2 × 10^5^ cells per dish. The fresh medium was replaced every other day. The ECs were used up to passage 5. To detach cells, 0.25% Trypsin-EDTA solution (Invitrogen) was used. To investigate the effect of microgravity, each petri dish with subconfluent ECs was mounted on a specific holder to insure nonexistence of any bubbles which possibly exert undesired shear on the cells. Total of 36 experiments were performed. Each experiment contains two independent samples; the test sample was under s-μg whereas the control one was maintained in the same cell culture incubator. A detail statistical analysis was performed and P < 0.05 was considered as a maximum to represent a statistically significant difference. ImageJ software was used to calculate the amount of circularity for the cells.

### Random Positioning Machine

**Figure 1:**
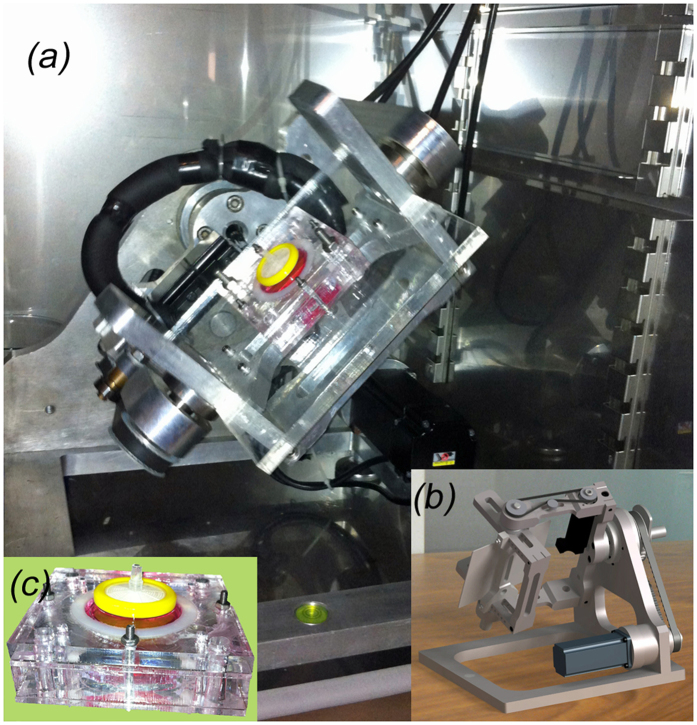
3D random positioning machine (RPM) for inducing simulated microgravity on ECs, (**a**) RPM setup was used to continuously make random changes in the orientation of ECs relative to the gravity vector. Each petri dish (35 mm in diameter) was inserted in a specific holder. A 22 μm-filter with 33 mm in diameter was inserted in the holder’s cap to provide air/medium exchange. (**b**) The schematic design of the RPM drawn using Autodesk Inventor (Autodesk Inc., San Rafael, California, USA). (**c**) Holder containing sample petri dish made by Poly(methyl methacrylate).

An RPM was developed to provide the s-μg by means of continuous random change of samples’ orientation relative to the gravity vector^19^,^52^. The machine contains two perpendicular frames controlled by a programmable logic controller (PLC) using two independent servomotors. The speed and rotation direction of each frame are determined based on random walk scenario^53^. To lessen the effect of cell-cell contact on mechanical properties of cells, non-confluent dishes were chosen for MA analysis. The cavity between petri dish and holder were completely filled with cell culture medium. Thereafter, a 22 μm filter was inserted in the cap to enable air/medium exchange and to maintain 5% CO_2_. Finally, the samples were mounted as close as possible to the center of the RPM which was initially located within the incubator (Fig. 1). The RPM functional parameters, random angular walk speed and the maximum distance of ECs to the center of rotation^52^, were adjusted to be 0.3–0.7 rad/s and 2 mm respectively. Before suspending the cells, ECs beyond the maximum distance were mechanically detached by cell scraper. This approach guaranteed the evaluated ECs to experience 10^−4 ^g as the maximum gravitational acceleration and 5 × 10^−4^ as the maximum tangential acceleration. The detailed calculation of the developed microgravity together with the data obained from a 3D acceleration sensor embedded in the test plate confirmed that the samples experience a gravity of less than 10^−4 ^g.

### Cell proliferation assay

MTT cell proliferation assay was used to evaluate the effect of microgravity on ECs proliferation. Briefly, after incubating for 24 hrs on normal conditions, one petri dish was mounted on the RPM stage utilizing the holder for another 24 hrs. Then, tetrazolium salt (M2128, Sigma) was added for an additional 4 hrs. Thereafter, formazan crystals were solubilized using Dimethyl sulfoxide (Sigma) while shaking for 20 min. Absorbance at 570 nm was measured by a standard spectrophotometer (CECIL, UK). The assay was performed six times for cells exposed to the s-μg condition for 24 hrs.

### Micropipette aspiration experiment

**Figure 2:**
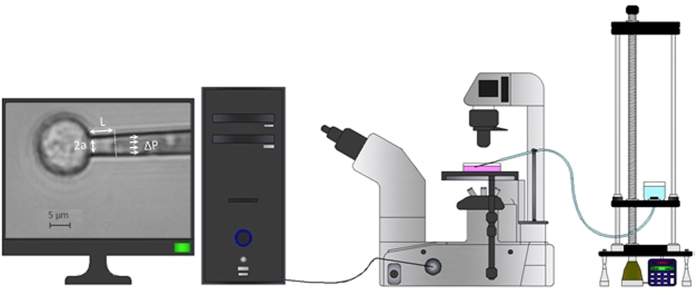
Micropipette aspiration technique was implemented for investigating the mechanical properties of ECs. Desired suction pressure is generated at the end of the micropipette by moving the water reservoir. Generalized Maxwell model’s parameters were estimated by dimensions, creep time, applied pressure, and aspirated length.

**Figure 3:**
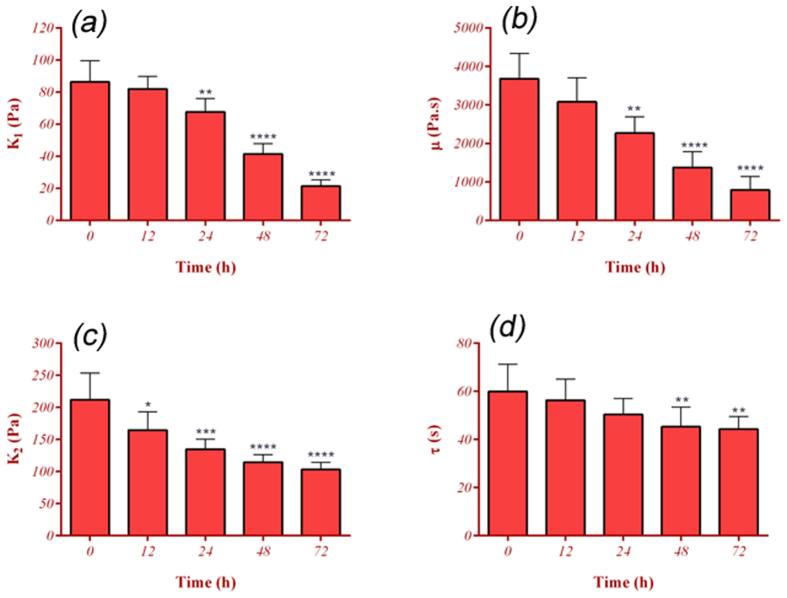
Alteration of viscoelastic parameters of ECs under microgravity condition. (**a**) Elastic constant, k_1_. (**b**) Elastic constant, k_2_. (**c**) Coefficient of viscosity, μ. (**d**) Time constant, τ. After 24 hrs being under microgravity simulation, a significant reduction in k_1_, k_2_ and μ were observed (^**^ describes statistical significance Of *p* < *0.01*). The alteration was much higher after 48 hrs (^****^ describes statistical significance Of *p* < *0.0001*). At least 36 cells (or 6 s-μg experiments) were studied for each time point. ^*^ and ^***^ indicate statistical significance of p < 0.05 and p < 0.001, respectively.

**Figure 4:**
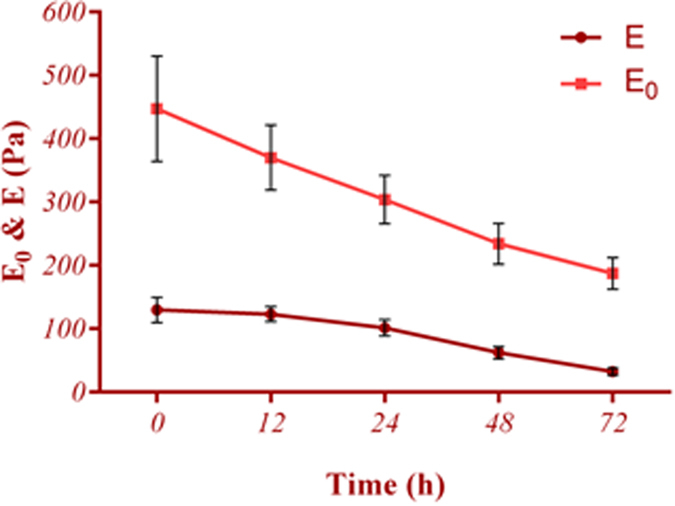
Young’s modulus (E) of ECs decreased smoothly whereas instantaneous modulus (E_0_) dropped dramatically even at short period of being under s-μg. E_0_ and E decreased approximately 20% and 5% at 12 hrs and reached 67% and 80% of the initial value at 24 hrs, respectively. Experiencing similar reduction of 50% at 48 hrs, the reduction percentage of Young’s modulus exceeds instantaneous Young’s modulus at 72 hrs. At least 36 cells were studied for each time point.

**Figure 5:**
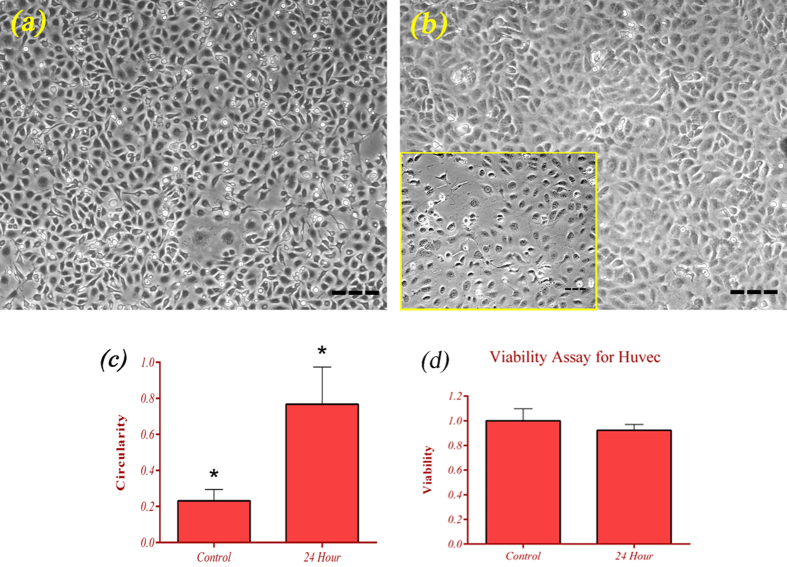
Morphology of ECs became more rounded subjected to microgravity. (**a**) HUVEC cells cultured in normal condition (scale bar represent 100 μm). (**b**) The cells found cobblestone phenotype after 24 hrs experiencing s-μg (scale bar represent 100 μm). The small yellow border image shows non-confluent petri dish (scale bar represent 50 μm). (**c**) Calculated circularity (the ratio of width to length) for ECs shows 3.3 times increase under s-μg and after 24 hrs compared to control cells. Six s-μg experiments were done to calculate the circularity parameter at this time point (n_cells_ > 800, p < 0.05). (**d**) Viability analysis at 24 hrs of s-μg showed no significant impact of microgravity condition on ECs.

**Figure 6:**
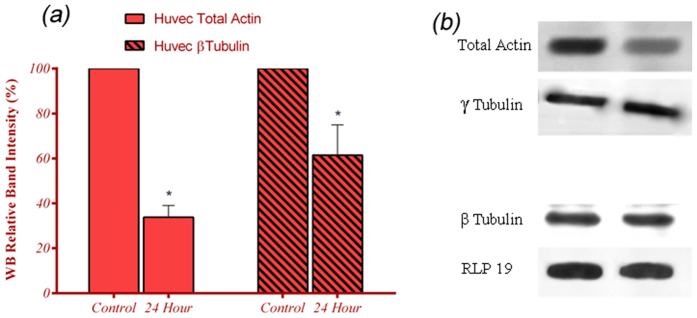
Western blot analysis revealed a considerable drop in the content of main cytoskeleton polymers. (**a**) Quantification of the proteins bands by ImageJ software revealed a difference between the amount of reductions in total actin and β-tubulin content of ECs caused by microgravity. (**b**) Whole cell extracts from cells in normal condition and cells exposed to s-μg for 24 hrs were subjected to western blot analysis with antibodies directed against actin filament and β-tubulin proteins. Expression levels are shown in percentage and normalized to RPL19 or γ-tubulin. Three biological replicates performed in duplicate ± the standard deviation (*p ≤ 0.05).

**Figure 7:**
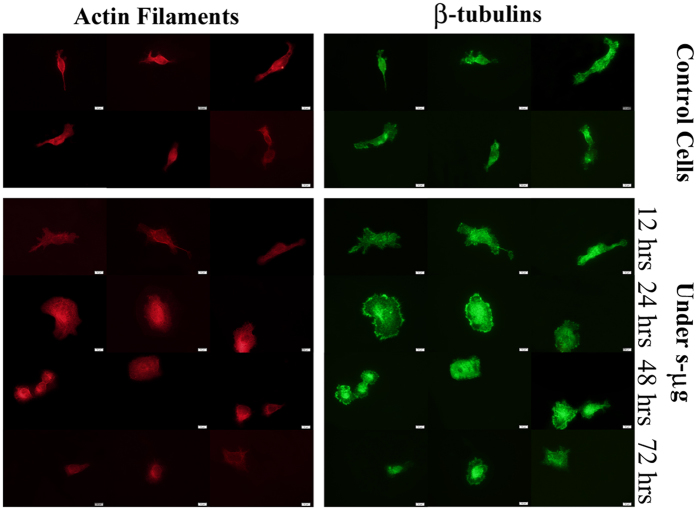
Fluorescent staining of ECs to visualize actin filaments (Fluorescein isothiocyanate labeled Phalloidin, red) and MTs (Monoclonal Anti-β-Tubulin-Cy3, green). Comparing with control cells, the more ECs subjected to simulated microgravity, the more their cytoskeleton disorganized. Scale bars represent 10 μm and all images were obtained with same magnification.

MA is a reproducible technique to quantify mechanical properties of cells. Considering appropriate mechanical models and subsequent theories, MA has been broadly utilized to estimate the whole body mechanical behavior of various cell types such as cancerous cells^24^,^54^, chondrocytes^55^, fibroblast^56^, and endothelial cells^14^. MA has also been used to investigate the alteration in skeletal and mechanical parameters of ECs as a consequence of external loadings^36^. Our developed MA arrangement for the present study is depicted in Fig. 2. The following procedure was performed according to the previous works^36^,^57^.

In brief, cells were suspended in the culture medium by 0.25% Trypsin-EDTA in less than 4 min to minimize the effect of trypsinization^58^. Furthermore, the measurement time for all samples was limited to less than 400 s to minimize the possible cytoskeletal reorganization after demounting from RPM machine. A movable water reservoir was implemented to apply the desired pressure at the end of the glass micropipette. Displacing the reservoir up or down leads to a suitable pressure to pull in or force away the cell, respectively. This is also applicable to find zero pressure. Actual pressure was related to the displacement of the reservoir provided by a precise motor. The step constant negative pressure imposed to each cell maintained to be between 500–600 Pa in all experiments. Micropipettes, with internal diameters within the range of 4.5–5 μm were coated with Sigmacote chemical agent (Sigma) to minimize the possible adhesion of the cells to the inner wall of the micropipettes^59^. The cell surface was aspirated into the pipette by exerting a controlled suction pressure on the cell body at room temperature around 20–22 °C. An inverted microscope equipped with a digital camera (Nikon Eclipse, DXM1200) provided monitoring of the leading edge of cell surface. Each micropipette was under control by a micromanipulator (Transfer Man Nk2, Eppendorf). At least three cells were investigated from each petri dish for MA study. Analysis of images was performed by Axiovision LE Software (Zeiss).

### Theoretical Model of Micropipette aspiration

Mechanical properties of cells in MA technique are extracted with a generalized Maxwell model. Cells are assumed to be a homogeneous, incompressible, and viscoelastic material subjected to a uniform axisymmetric aspiration pressure as depicted in Fig. 2. The model includes three constants: a spring (k_1_) that provides the restoring force necessary to recover the initial shape after the release of the stress in parallel with serially arranging of a damper (μ) and a spring (k_2_)^36^. The relation between elastic and viscous parameters is described by Equ. 1 in which the boundary condition of no axial displacement of the cell is assumed at the micropipette end.




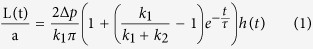




where *∆P* is the applied pressure, *L*(*t*) is the aspirated length, *h*(*t*) is the unit step function, and *a* is considered as the inner radius of the micropipette. The time constant (*τ*) of viscoelastic model is calculated by Equ. 2:




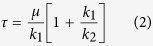




Given the following equations, viscoelastic parameters of cells are obtained by curve fitting of experimental data (*L/a*) with time using the least square method implemented in Matlab:




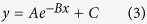







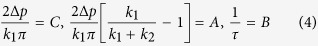




### Fluorescence labeling for microscopy

Cells were seeded to proliferate for 24 hrs on sterile cover slips. The cover slips were then mounted on the simulating microgravity machine for another 24 hrs. Fluorescein isothiocyanate labeled Phalloidin (Sigma, P5282) was used to visualize actin filaments. The staining procedure started immediately after each s-μg experiment. After several times rinsing with phosphate buffered saline (PBS), the cells were fixed with 3.7% paraformaldehyde (dissolved in PBS buffer) for 5 min. Upon permeabilizing cells with 0.1% Triton-X100 in PBS and washing again in PBS, they were stained with a 50 mg/ml fluorescent phalloidin conjugate solution in PBS for 40 min at room temperature. Before staining for microtubules, samples were washed several times with PBS to remove unbound phalloidin conjugate. Detection and localization of microtubules were performed using monoclonal Anti-β-Tubulin-Cy3 (Sigma, C4585). Diluted antibody conjugate in PBS containing 1% BSA was added to cover cell layer and incubated for 60 min. Upon 3 times washing of the cells with PBS, 5 minutes each, samples were left to dry. Images were captured with an invert fluorescent microscope (Olympus, BX51 equipped with DP72 camera) and processed with ImageJ software.

### Western Blot Analysis

Western Blot analysis was performed immediately after simulated microgravity experiments to quantify the content of the major cytoskeleton polymers^60^. Whole cells were lysed by RIPA buffer added with 1x complete protease inhibitor cocktail tablet (Roche). Total protein concentration was determined by the Bradford method (Bio-Rad protein assay). After electrophoresis, proteins were transferred to Methanol pre-wetted polyvinylidene difluoride (PVDF) membranes (Millipore). Membranes were blocked with 2% ECL Advance blocking agent (Amersham) in PBS-Tween. Mouse monoclonal antibodies targeting total actin and β-tubulin (Sigma) were diluted in the blocking solution and incubated over night at 4 °C. Detection of bound antibodies was performed with peroxidase coupled secondary antibodies using the ECL Advance Western blotting detection system (Amersham). The bands obtained on X-ray films were quantified using the NIH ImageJ program. Expression of actin and β-tubulin content are presented in the average of three biological replicates. To ensure equal loading, ribosomal protein L19 or γ–tubulin (Sigma–Aldrich) was used.

### Statistical analysis

The analysis of variance (ANOVA) followed by Dunnet post-test was done to compare the results. The Dunnet post-test decreases considerably the number of comparisons and increases the power for detecting the differences. This method compares each test group with the control instead of involving nonrelated groups. All data are presented as mean ± standard error of the mean. All statistical analysis and the related graphs were performed in GraphPad Prism 6.0. (CA, USA). P < 0.05 was considered as a maximum to represent a statistically significant difference.

## Additional Information

**How to cite this article**: Janmaleki, M. *et al.* Impact of Simulated Microgravity on Cytoskeleton and Viscoelastic Properties of Endothelial Cell. *Sci. Rep.* **6**, 32418; doi: 10.1038/srep32418 (2016).
